# Domain Shift Analysis in Chest Radiographs Classification in a Veterans Healthcare Administration Population

**DOI:** 10.1007/s10278-025-01494-7

**Published:** 2025-04-11

**Authors:** Mayanka Chandrashekar, Ian Goethert, Md Inzamam Ul Haque, Benjamin McMahon, Sayera Dhaubhadel, Kathryn Knight, Joseph Erdos, Donna Reagan, Caroline Taylor, Peter Kuzmak, John Michael Gaziano, Eileen McAllister, Lauren Costa, Yuk-Lam Ho, Kelly Cho, Suzanne Tamang, Samah Fodeh-Jarad, Olga S. Ovchinnikova, Amy C. Justice, Jacob Hinkle, Ioana Danciu

**Affiliations:** 1 Advanced Computing for Health Sciences, Computational Sciences and Engineering Division, Oak Ridge National Laboratory, Oak Ridge, TN USA; 2 IT Services Division, Oak Ridge National Laboratory, Oak Ridge, TN USA; 3 Thermo-Fisher Scientific, Waltham, MA USA; 4 Theoretical Biology Group, Los Alamos National Laboratory, Los Alamos, USA; 5 Department of Veterans Affairs, West Haven, CT USA; 6 Yale School of Medicine, New Haven, CT USA; 7 Division of Aging, Brigham and Women’s Hospital, Boston, MA USA; 8 Department of Medicine, Harvard Medical School, Boston, MA USA; 9 Million Veteran Program Boston Coordinating Center, VA Boston Healthcare System, Boston, MA USA; 10 Division of Immunology and Rheumatology, Department of Medicine, Stanford University, Stanford, CA USA; 11 Department of Veterans Affairs, Office of Mental Health and Suicide Prevention, Program Evaluation Resource Center, Palo Alto, CA USA; 12 NVIDIA corporation, Santa Clara, CA USA; 13 Department of Biomedical Informatics, Vanderbilt University Medical Center, Nashville, TN USA

**Keywords:** Domain shift, Chest X-ray image classification, Multi-label classification

## Abstract

This study aims to assess the impact of domain shift on chest X-ray classification accuracy and to analyze the influence of ground truth label quality and demographic factors such as age group, sex, and study year. We used a DenseNet121 model pre-trained MIMIC-CXR dataset for deep learning-based multi-label classification using ground truth labels from radiology reports extracted using the CheXpert and CheXbert Labeler. We compared the performance of the 14 chest X-ray labels on the MIMIC-CXR and Veterans Healthcare Administration chest X-ray dataset (VA-CXR). The validation of ground truth and the assessment of multi-label classification performance across various NLP extraction tools revealed that the VA-CXR dataset exhibited lower disagreement rates than the MIMIC-CXR datasets. Additionally, there were notable differences in AUC scores between models utilizing CheXpert and CheXbert. When evaluating multi-label classification performance across different datasets, minimal domain shift was observed in the unseen VA dataset, except for the label “Enlarged Cardiomediastinum.” The subgroup with the most significant variations in multi-label classification performance was study year. These findings underscore the importance of considering domain shift in chest X-ray classification tasks, paying particular attention to the temporality of the exam. Our study reveals the significant impact of domain shift and demographic factors on chest X-ray classification, emphasizing the need for improved transfer learning and robust model development. Addressing these challenges is crucial for advancing medical imaging research and improving patient care.

## Introduction

Chest radiography is the first-line imaging test for respiratory and some forms of cardiac disease. Chest X-ray abnormalities detection has been automated by recent advanced artificial intelligence techniques [1, 2]. Accurate chest X-ray classification has played an important role in many biomedical applications to accelerate the diagnosis and treatment of conditions like pneumonia, heart failure, rib trauma, pulmonary fibrosis, etc. [3]. Although machine learning models show great promise to enhance diagnostic capabilities, the efficacy of these models hinges on the availability and quality of training data. [4].

Recent years have seen a wave of artificial intelligence (AI) and machine learning models for clinical applications trained on data from large research medical centers or based on limited de-identified datasets [5]. The introduction of transfer learning has resulted in AI models being accessible to researchers with fewer computational resources and less data, allowing them to leverage existing knowledge. Transfer learning is the ability to apply a model trained on a dataset to another dataset of interest. A major consideration for transfer learning is domain shift, the dissimilarity between data distributions from the source used for training and the population to which the models are applied. This divergence between open datasets and private, institution-specific datasets can introduce substantial bias and hinder the generalization of machine learning models to real-world scenarios [6]. The central challenge lies in ensuring the generalizability of machine learning models across diverse clinical datasets. The existence of domain shifts due to differences in patient demographics, imaging protocols, and annotation variability significantly limits the effectiveness of pre-trained models in real-world settings. This study addresses these gaps by systematically quantifying the impact of domain shifts and proposing strategies to mitigate their effects, ensuring robust deployment of AI tools in healthcare.

In this study, we quantify the domain shift between the open domain (MIMIC-CXR) and a private dataset (VA-CXR). The efficacy of the transfer learning approach for chest X-ray classification and the subsequent impact on classification accuracy when dealing with domain shift form the main focus of this study. The domain shift is traditionally viewed only based on the model and its performance, ignoring the multi-fold causes leading to the shift. We comprehensively address the effects of domain shift in this three-stage study: (1) Compare the performance between the source domain and target domain accuracy on the chest X-ray classification. (2) Quantify the quality of the ground truth extracted from the radiology reports, as supervised learning models are heavily dependent on the quality of the labels. (3) Analyze the relationship between demographic factors and classification accuracy, as domain mismatches often originate in demographic mismatches.

**Table 1:** Source and target dataset

	Source dataset		Target dataset	
Name	MIMIC-CXR		VA-CXR	
Availability	Open		Private	
Clinical setting	Inpatient		Outpatient	
Time	2011 to 2016		2010 to 2022	
# Images	377,110		259,361	
# Studies	227,827		91,020	
# Patients	65,379		35,771	
# Studies by age (percentage %)				
(0–50]	51,607	(22.65)	8589	(9.43)
(50–60]	41,661	(18.29)	12,258	(13.46)
(60–70]	51,168	(22.46)	30,736	(33.76)
(70–80]	43,060	(18.9)	23,360	(25.66)
(80–90]	30,854	(13.54)	13,229	(14.53)
(90–100]	9477	(4.16)	2825	(3.10)
#Patients by sex (percentage %)				
Male	34,127	(52.2)	33,381	(93.31)
Female	31,006	(47.4)	2,390	(6.68)
NaN	246	(3.76)	–	–

By addressing the critical interplay of domain shift and demographic factors, this study not only provides insights into the technical challenges of adapting machine learning models to private datasets but also underscores the broader implications of these challenges in the context of healthcare. Our research contributes to developing more accurate, robust, and generalizable chest X-ray classification models by creating a systematic approach for using an existing model and understanding the nuances of a model’s performance before applying it to a new population. To further highlight our study’s innovations, we emphasize the systematic approach to evaluating domain shift by integrating three distinct dimensions: ground truth label quality, demographic influences, and subgroup analyses such as study year and sex. Unlike prior works [4, 7], which focus primarily on model performance, our study uniquely provides a comprehensive understanding of domain shift in real-world medical imaging datasets and offers practical insights for transfer learning applications in medical applications.

**Fig. 1:**
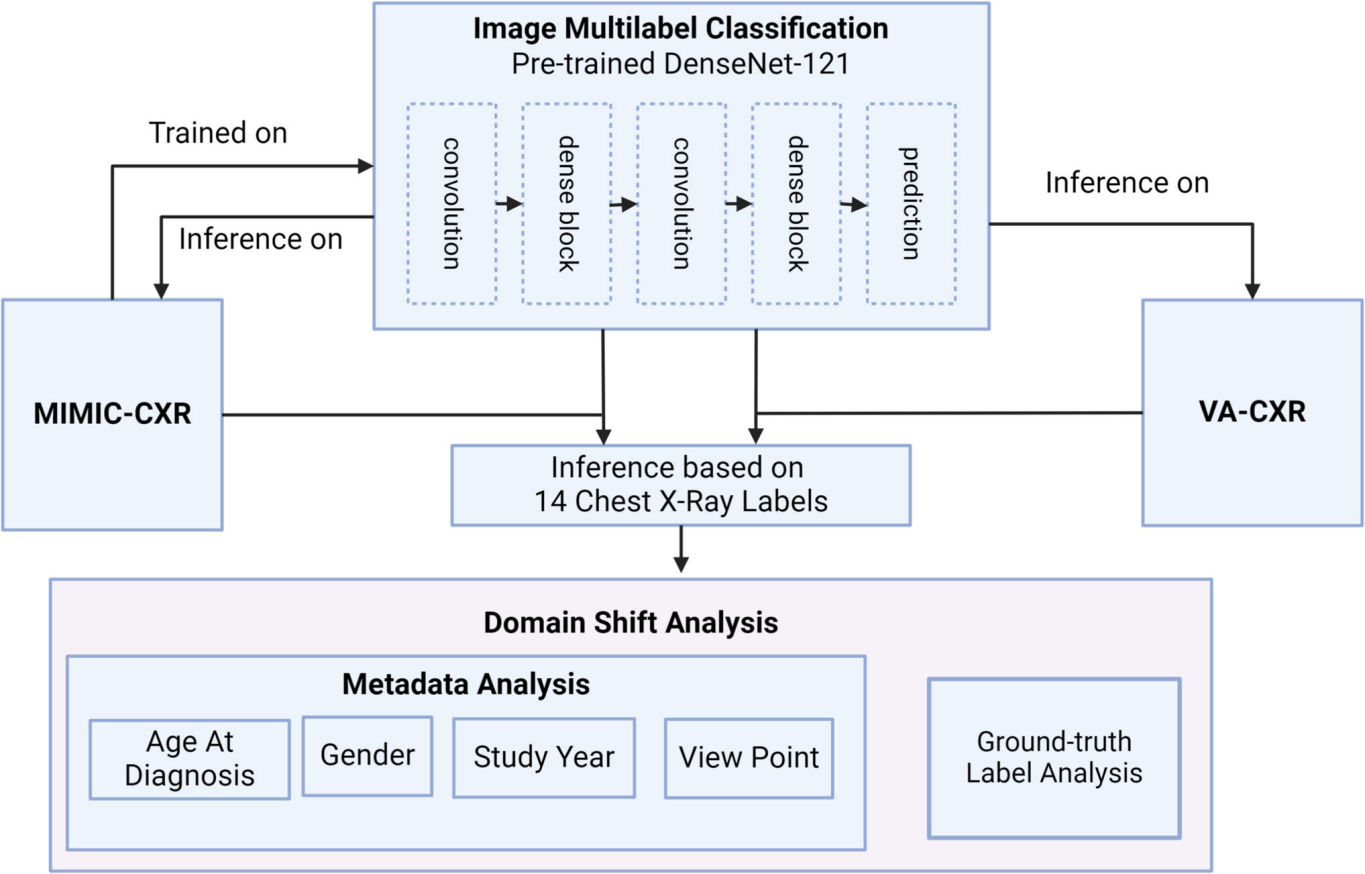
Domain shift analysis based on image classification model

## Methods

### Datasets

#### MIMIC-CXR

is an open dataset consisting of 377,110 chest X-rays associated with 227,827 imaging studies from 65,379 patients. MIMIC-CXR is collected from the inpatient setting of Beth Israel Deaconess, a Boston hospital (refer Table 1). The test split of this dataset (Test Split MIMIC-CXR), which consists of 5159 studies with 293 patients, served as the hold-out set analysis [8].

#### VA-CXR

is a private dataset of 259,361 chest X-rays associated with 91,020 imaging studies from 35,771 patients. VA-CXR is collected in the outpatient setting of the Boston Veterans Healthcare Administration station (refer to Table 1). The ground truth labels were extracted from the VA’s corporate data warehouse (CDW) and joined with images using patient information from DICOM headers as published in Knight et al. [23].

### Ground Truth Label Extraction

We used the CheXbert labelers [9] to expertly assign labels to 14 specific labels (atelectasis, cardiomegaly, consolidation, edema, enlarged cardiomediastinum, fracture, lung lesion, lung opacity, no finding, pleural effusion, pleural other, pneumonia, pneumothorax, support devices) associated with different chest conditions from radiology reports. CheXbert is a BERT-based approach that automates the detection of these observations, effectively streamlining the process of annotating medical images and reports. The NLP label extraction outputs scores for four classes: positive, negative, blank, and uncertain, associated with each of the 14 labels. As the class names indicate, for example, for *pneumonia*, positive class: radiology report indicates that the patient has *pneumonia*; negative class: radiology report indicates that the patient does not have *pneumonia*; uncertain class: radiology report mentions *pneumonia*, but the NLP tool is unable to determine if it is positive or negative; blank class: radiology report does not mention *pneumonia*.

### Label Validation

Because ground truth assignment ultimately determines the accuracy of the imaging classifiers, we developed an image evaluation procedure in two steps. First, we evaluated the agreement between the NLP label extraction tool, CheXbert, and its precursor, CheXpert [10], a rule-based tool. We focused on positive class agreements for our evaluation, using only positive classes for classification, and have combined uncertain/negative class agreements. The disagreement between NLP label extraction tools indicates ambiguity/less confidence on the labels, ultimately creating an unreliable ground truth. The agreement is measured for both MIMIC-CXR and VA-CXR datasets. Of note that neither of the datasets was used to train the NLP extraction tools.

#### Relation to Diagnoses Codes

To validate the ground extracted from CheXpert-labeler, we analyzed the relationship of specific ground truth labels to ICD codes in the patient’s electronic health record (EHR) extracted from the VA’s Corporate Data Warehouse (CDW). The assignment of ICD-9 and ICD-10 diagnosis codes associated with each condition was exploratory and not extensively optimized.

Starting concepts were retrieved from the CheXpert-labeler github repository, where phrases they used to search notes can be found at *https://github.com/stanfordmlgroup*. These phrases, along with our own expertise, were used to identify diagnosis codes and cross-reference radiology reports with diagnoses. For example, pneumonia was identified by CheXpert-labeler as indicated by *pneumonia*, *infection*, *infected process*, and *infectious*; edema was indicated by terms *edema*, *heart failure*, *chf*, *vascular congestion*, *pulmonary congestion*, *indistinctness*, and *vascular prominence*; fracture was indicated solely by the word *fracture*; and pneumothorax was identified by either *pneumothorax* or *pneumothoraces*.

This method enabled us to validate the ground truth labels by correlating them with the relevant ICD codes in the patients’ EHRs, ensuring accurate cross-referencing of radiology reports with diagnoses.

For instance, the ICD-9 codes we used to indicate a pneumonia diagnosis in the outpatient diagnosis tables ranged from 480 to 486 and included 487.0. These codes encompass viral, bacterial, and other types of pneumonia, as well as pneumonia caused by unspecified pathogens. A similar approach was applied to ICD-10 codes for pneumonia and other conditions. The specific ICD codes used for each condition are detailed in Appendix Table 6.

### Multi-Label Image Classification

Using the 14 labels extracted from the corresponding radiology reports with CheXbert, we created a multi-label image classification model from X-ray images (as shown in Fig. 1). We used a pre-trained DenseNet model [11] as the core framework, removed the top classification layer, and integrated a custom classification layer for multi-label output. Previous work shows the effectiveness of different resolutions of DenseNet121-based multi-label classification chest X-ray model on MIMIC-CXR dataset [12]. This work uses the MIMIC-CXR trained DenseNet-121 model on chest X-ray pre-processed into 256x256 JPG images [8, 13]. We also compare the MIMIC-CXR trained DenseNet-121 model with category-wise fine-tuning (CFT) [14]. CFT model is trained based on CheXpert dataset [10], and it was listed as the best performing model on CheXpert as of Jan ’24 [15].

### Metrics

We evaluated our models using the area under the curve (AUC). The AUC score was calculated separately for each of the 14 labels, indicating the separability measure for a given chest X-ray label. We also analyzed the difference in AUC scores between MIMIC-CXR and VA-CXR, and the prevalence for each label was calculated as the number of studies with positive results for a given label divided by the total number of labels, indicating the label’s presence in the given cohort.

**Table 2:** Groundtruth analysis: agreement and disagreement rates between CheXpert and CheXbert across 14 classification labels

Labels	MIMIC-CXR						VA-CXR					
	Positive agreement		U/N* agreement		Disagreement		Positive agreement		U/N* agreement		Disagreement	
	Count	%	Count	%	Count	%	Count	%	Count	%	Count	%
Atelectasis	44,422	19.5	8557	3.76	23,028	10.1	8902	9.8	4454	4.9	621	0.7
Cardiomegaly	35,733	15.7	19,798	8.69	88,995	39.1	8437	9.3	29,427	32.3	12,459	13.7
Consolidation	9785	4.3	10,868	4.77	54,675	24.0	1306	1.4	24,019	26.4	4863	5.3
Edema	25,559	11.2	32,134	14.10	39,799	17.5	2104	2.3	35,534	39.0	2864	3.1
ECM	4612	2.0	13,365	5.87	95,973	42.1	2487	2.7	27,615	30.3	14,264	15.7
Fracture	3920	1.7	797	0.35	10,941	4.8	4049	4.4	677	0.7	588	0.6
Lung lesion	5769	2.5	973	0.43	8877	3.9	5408	5.9	1497	1.6	2295	2.5
Lung opacity	44,982	19.7	2065	0.91	30,895	13.6	16,965	18.6	9696	10.7	19,734	21.7
No finding	18,951	8.3	0	0.00	56,921	25.0	12,654	13.9	0	0.0	3770	4.1
PE	52,084	22.9	28,513	12.52	100,762	44.2	11,804	13.0	59,301	65.2	4729	5.2
PO	1960	0.9	645	0.28	3761	1.7	4192	4.6	544	0.6	959	1.1
Pneumonia	8054	3.5	36,599	16.06	40,233	17.7	2351	2.6	3913	4.3	3083	3.4
PT	8461	3.7	41,010	18.00	100,543	44.1	3344	3.7	34,191	37.6	2780	3.1
SD	63,526	27.9	827	0.36	28,196	12.4	13,294	14.6	233	0.3	8221	9.0

$*$Uncertain/negative; *PE*, pleural effusion; *PT*, pneumothorax; *SD*, support devices; *PO*, pleural other;*ECM, enlarged cardiomediastinum

### Domain Shift Analysis

We compared our source and target datasets along different dimensions: (1) Demographic details: age at time of imaging study, sex; (2) imaging study details: study year, view point (lateral view (Lat), erect anteroposterior (AP), posteroanterior (PA)); (3) ground truth labels: 14 labels. All the factors were analyzed against the accuracy of multi-label image classification. The impact can be estimated by comparing the prevalence of the above-listed factors across source and target domains with the performance of the multi-label classification. The subgroup analysis is performed on unseen datasets: Test Split MIMIC-CXR and VA-CXR.

#### Study Year Analysis

We obtain the study year from VA-CXR studies date reported in DICOM headers. The MIMIC-CXR dataset is unidentified; study years are replaced by anchor years. The comparison of performance across study years between the datasets is not possible, so we do not present a study year analysis on the MIMIC-CXR dataset.

#### Performance Analysis Based on Sex

We obtain the sex of the patients in VA-CXR from the VA’s corporate data warehouse (CDW) and the patients in MIMIC-CXR from the metadata. Sex is only classified into binary classes of male and female. We analyze the label-wise performance for each sex across both datasets.

#### View Position Analysis

We obtain the view position of the images from the DICOM metadata. We use four main viewpoints for analysis: lateral view (Lat), erect anteroposterior (AP), posteroanterior (PA)) and left lateral view (LL). We compare the label-wise performance across both datasets

#### Age Group Analysis

We define the age of the patient as the age when the imaging study was performed. For VA-CXR, this age was calculated from the date of birth obtained from VA CDW and the date of imaging study from DICOM metadata. For MIMIC-CXR, we calculated age using the anchored date of birth and anchored imaging study date. The anchored dates were amended in a manner that the difference in years is constant, so we were able to approximate the age of the patient. The label-wise performance for both datasets was compared across six age groups: (0–50], (50–60], (60,70], (70,80], (80,90], and (90,100].

## Results

### Ground Truth Label Validation

Table 2 presents a comprehensive analysis of agreement and disagreement rates between CheXpert and CheXbert on the MIMIC-CXR and VA-CXR datasets across the 14 labels. The results shed light on the performance and consistency of CheXpert and CheXbert and the uncertainty of the ground truth labels. Notably, in MIMIC-CXR, atelectasis was identified in 19.5% of cases with a disagreement rate of 10.1%, whereas in VA-CXR, the identification rate was lower at 9.8% with a disagreement rate of only 0.7%. This discrepancy in positive identification and disagreement rates is further exemplified in conditions like cardiomegaly, where MIMIC-CXR reported positive identification in 15.7% of cases with a significant disagreement rate of 39.1%, contrasting with VA-CXR’s 9.3% positive identification rate and 13.7% disagreement rate.

**Fig. 2:**
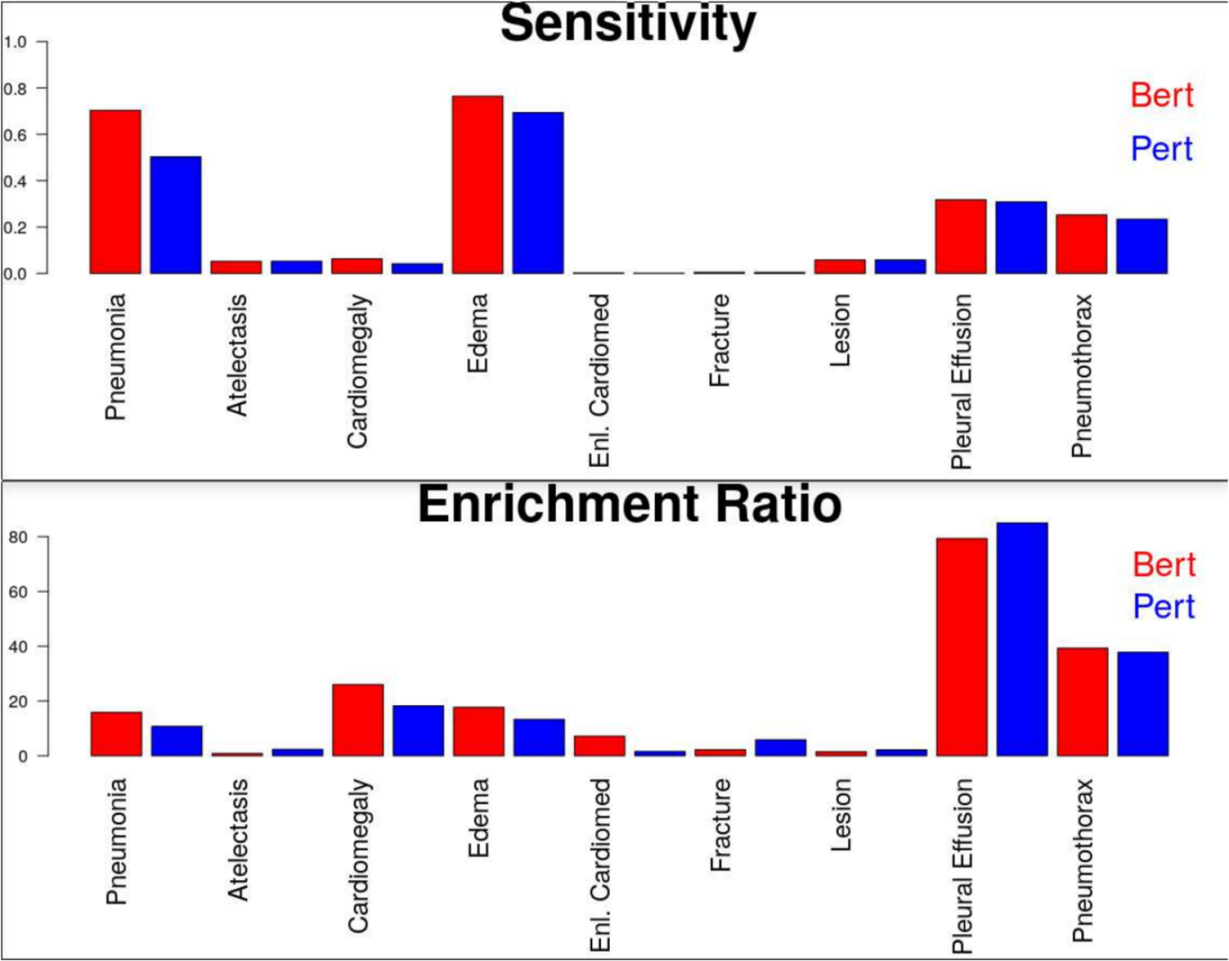
Characterization of the concurrence of positive findings on chest X-rays with associated diagnoses for our VA data set. The top panel shows the sensitivity with which a related diagnosis is given within a week before or after a positive X-ray finding, which we labeled “sensitivity.” The bottom panel shows the factor by which the ratio of positive to negative X-ray findings increases when a diagnosis code is present. Patients with a diagnosis code not within 1 week of the assessed X-ray are excluded from the calculation for both plots. Bert, CheXbert model; Pert, CheXpert model

**Table 3:** Multi-label image classification using MIMIC-CXR (plus CheXpert) as source. Results shown in table show the performance of VA-CXR on two NLP ground truth extraction for VA-CXR

	CheXpert				CheXbert			
**Finding**	DN*	CFT*	Prevalence	Count	DN*	CFT*	Prevalence	Count
Atelectasis	0.801	0.788	0.101	21,329	0.802	0.789	0.100	21,169
Cardiomegaly	0.753	0.742	0.149	31,461	0.862	0.853	0.100	21,151
Consolidation	0.746	0.752	0.025	5265	0.811	0.809	0.016	3308
Edema	0.794	0.784	0.035	7453	0.854	0.836	0.026	5528
ECM*	0.519	0.549	0.161	33,970	0.600	0.640	0.035	7466
Fracture	0.619	0.611	0.047	9910	0.622	0.611	0.046	9623
Lung lesion	0.647	0.643	0.066	13,975	0.652	0.647	0.069	14,609
Lung opacity	0.716	0.712	0.260	54,872	0.736	0.745	0.199	41,965
No finding	0.784	0.787	0.165	34,771	0.790	0.795	0.156	32,883
Pleural effusion	0.920	0.908	0.136	28,770	0.927	0.917	0.141	29,689
Pleural other	0.746	0.707	0.046	9806	0.739	0.700	0.055	11,703
Pneumonia	0.683	0.661	0.055	11,533	0.755	0.727	0.030	6306
Pneumothorax	0.868	0.860	0.043	8973	0.898	0.891	0.037	7845
Support devices	0.793	0.809	0.211	44,596	0.804	0.808	0.161	33,935

$*$*ECM*, enlarged cardiomediastinum; *DN*, DenseNet121; *CFT*, category-wise fine-tuning

### Comparison with Diagnosis Codes

While the X-ray classification accuracy was evaluated against the label extracted using NLP of the appropriate radiology report, it is possible also to compare directly against diagnosis codes in the clinical record. However, interpreting this comparison is challenging due to two main issues: first, diagnoses are based on a broader range of information beyond just the X-ray, and second, patients may have multiple conditions, making it difficult to determine the specific reason each X-ray was ordered. Despite these challenges, we have conducted two useful comparisons of X-ray findings against diagnosis codes for nine categories within our dataset.

The top of Fig. 2 shows the fraction where a diagnosis related to the label was recorded within a week (either before or after) of the X-ray, compared to the total number of patients, including those who never had the diagnosis. Patients who had the diagnosis but not within the 1-week window were excluded from this calculation. This plot, labeled “sensitivity,” reflects the proportion of cases where a diagnosis occurs within a week of noting the condition in the X-ray. We observe a positive finding on an X-ray, as extracted from the radiology report, which is mostly associated with a specific diagnosis of pneumonia and edema and frequently associated with pleural effusion and pneumothorax. We also see that the CheXbert model is more frequently associated with a corresponding diagnosis than the CheXpert model.

**Table 4:** Comparison of label-wise accuracy between source, hold-out source, and target datasets

Finding	MIMIC-CXR		Test split MIMIC-CXR		VA-CXR		AUC Difference between hold-out and target (*P*-value)
	Source dataset		Hold-out source dataset		Target dataset		
	AUC	Count	AUC	Count	AUC	Count	
**Atelectasis**	0.808	45,808	0.762	1034	0.801	21,329	$-$0.039 (0.108)
Cardiomegaly	0.816	44,845	0.793	1258	0.753	31,461	0.04 (0.078)
Consolidation	0.821	10,778	0.762	326	0.746	5265	0.016 (0.712)
Edema	0.889	27,018	0.832	959	0.794	7453	0.038 (0.180)
ECM*	0.739	7179	0.727	200	0.519	33,970	0.208 (5.34E-5)
Fracture	0.667	4390	0.714	167	0.619	9910	0.095 (0.087)
Lung lesion	0.738	6284	0.722	202	0.647	13,975	0.075 (0.142)
Lung opacity	0.749	51,525	0.705	1561	0.716	54,872	$-$0.011 (0.543)
No finding	0.853	75,455	0.809	983	0.784	34,771	0.025 (0.338)
Pleural effusion	0.919	54,300	0.894	1542	0.920	28,770	$-$0.026 (0.266)
Pleural other	0.806	2011	0.806	119	0.746	9806	0.06 (0.419)
Pneumonia	0.714	16,556	0.713	539	0.683	11,533	0.03 (0.338)
Pneumothorax	0.856	10,358	0.813	144	0.868	8973	$-$0.055 (0.421)
Support devices	0.898	66,558	0.876	1457	0.793	44,596	0.083 (3.58E-4)

$$*$$Enlarged cardiomediastinum

The bottom of Fig. 2 compares the factor by which the enrichment ratio of positive finding in the radiology report increases with a concurrent (within 1 week) diagnosis. Pleural effusion, for example, is seen 10 times more frequently in the radiology report when a concurrent diagnosis code is noted. It is also true, however, that 2/3 of the pleural effusion diagnoses are not accompanied by a positive finding in a radiology report, as shown in the top bar chart, and also that 90% of the time a negative finding is made, no diagnosis is found. We see that the conditions with the highest enrichment ratios differ from those with the highest sensitivities, but the trend that the CheXbert model generally outperforms the CheXpert model by a few to 20% remains.

Several factors were observed to contribute to the quantitative comparison of labels extracted from the radiology reports to the observed diagnoses, including the extent to which diagnosis codes could be found that correspond well to the radiology report finding, whether the X-ray is a screening or confirmatory test, the prevalence of the condition, and the chronic vs. acute nature of the condition. We provide this figure as a survey across these factors and present the complete set of counts in Appendix Table 7. Specific ICD 9 and 10 codes used for each condition are provided in the Appendix Table 6.

### Multi-Label Image Classification on VA-CXR Across NLP Tools

We evaluated the label-wise performance of the VA-CXR dataset using CheXpert and CheXbert with DenseNet and category-wise fine-tuning (CFT) models (shown in Table 3). Across the 14 labels, CheXbert generally achieved higher AUC scores, particularly for critical findings like cardiomegaly (0.862 with DenseNet) compared to CheXpert (0.753). This trend highlights CheXbert’s superior robustness for complex conditions. However, for some labels, such as fracture, AUC differences between CheXpert and CheXbert were negligible ($\tilde{0}$.62), indicating that both tools perform similarly for specific tasks. Labels with higher prevalence, such as pleural effusion and support devices, consistently achieved strong AUC scores (e.g., pleural effusion: 0.920 with DenseNet), while challenging labels like enlarged cardiomediastinum (ECM) showed significantly lower AUC scores (0.519 for DenseNet with CheXpert), likely due to their lower prevalence and inherent diagnostic difficulty.

When comparing model architectures, DenseNet marginally outperformed CFT for high-prevalence labels like pleural effusion, while CFT showed slightly better performance for underrepresented labels such as ECM (0.640 with CheXbert). Prevalence and count discrepancies between CheXpert and CheXbert (e.g., lung opacity prevalence: 26.0% vs. 19.9%) also contributed to performance variations. These results emphasize the importance of selecting appropriate NLP tools and models for specific classification tasks and underscore the interplay between label prevalence and model performance, particularly for low-prevalence or diagnostically complex conditions.

### Multi-Label Image Classification Performance Across Multiple Datasets

Table 4 shows the comparison of MIMIC-CXR (source dataset), Test Split MIMIC-CXR (hold-out source dataset), and VA-CXR based on AUC on the 14 labels. The hold-out source dataset is the test split of MIMIC-CXR dataset, DenseNet-121 model. The test split of MIMIC-CXR gives us a fair comparison to VA-CXR, the unseen target dataset. This can be observed based on the AUC drop from the overall MIMIC-CXR to test split. In Table 4, the difference in AUC between hold-out and target indicates the performance variation between VA-CXR and test split of MIMIC. The negative value of the difference in AUC indicates that the VA-CXR performs better than the Test Split MIMIC-CXR, and the positive value indicates that the test split performs better. The enlarged cardiomediastinum (ECM) label has the highest difference in AUC, indicating a huge performance drop in VA-CXR. This could directly impact the lack of a large number of image studies in ECM in the source dataset compared to the target. Based on the *p*-value calculated using the AUC two-tailed z-test, it was observed that ECM and support devices show stastically significant difference between MIMIC-CXR and VA-CXR.

### Study Year-Wise Performance on VA-CXR

**Fig. 3:**
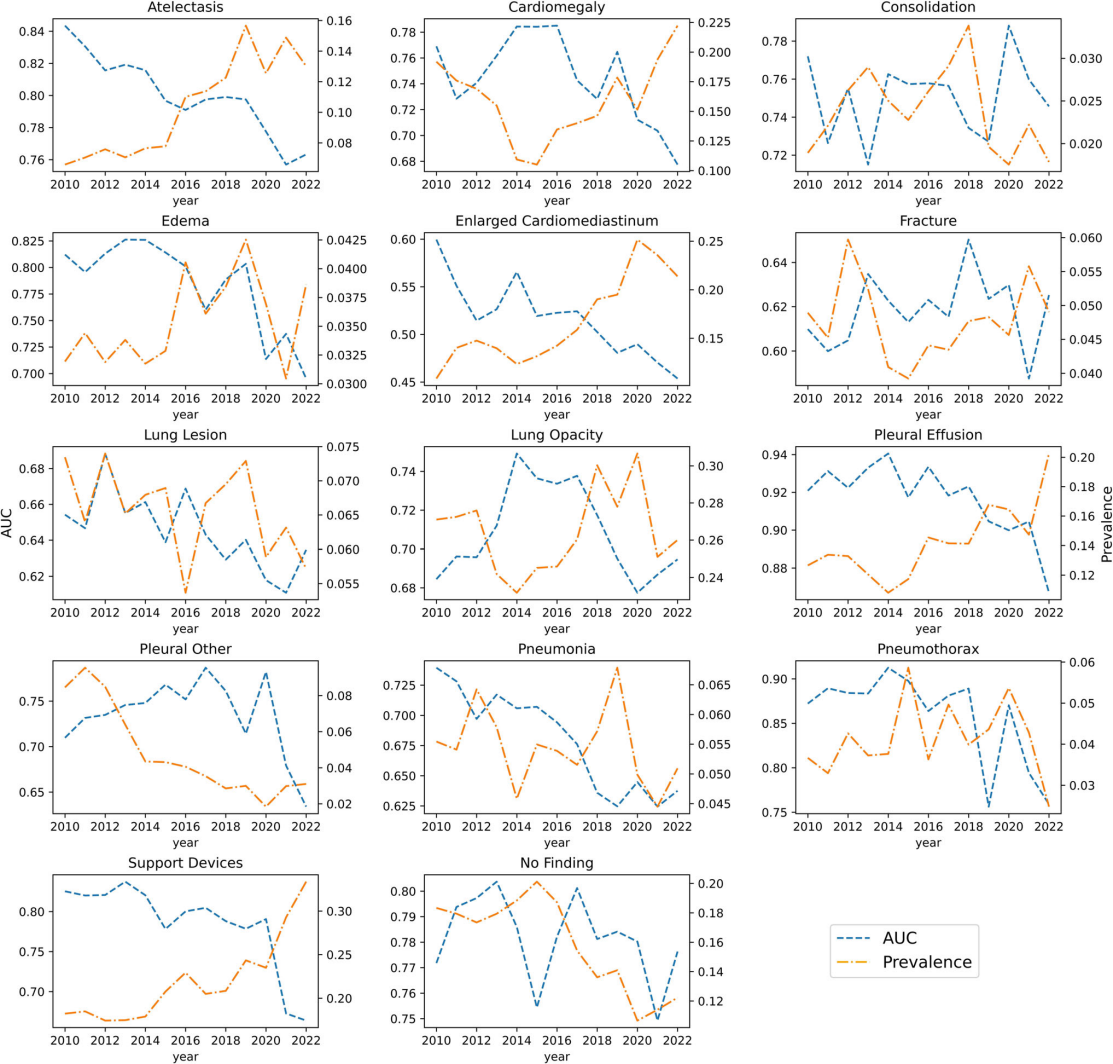
Accuracy and prevalence of labels study year wise. $*$Blue line indicates the AUC across years. Orange line indicates prevalence across years

Figure 3 shows the label-wise distribution of the study years. The AUC systematically drops as for study year *2020 to 2022* for all labels except *consolidation* and *pleural other*, which peaks at *2020*. The prevalence increases over the years in VA-CXR for *atelectasis*, *enlarged cardiomediastinum*, and *pleural effusion*.

### Performance Across Datasets Based on Sex

**Fig. 4:**
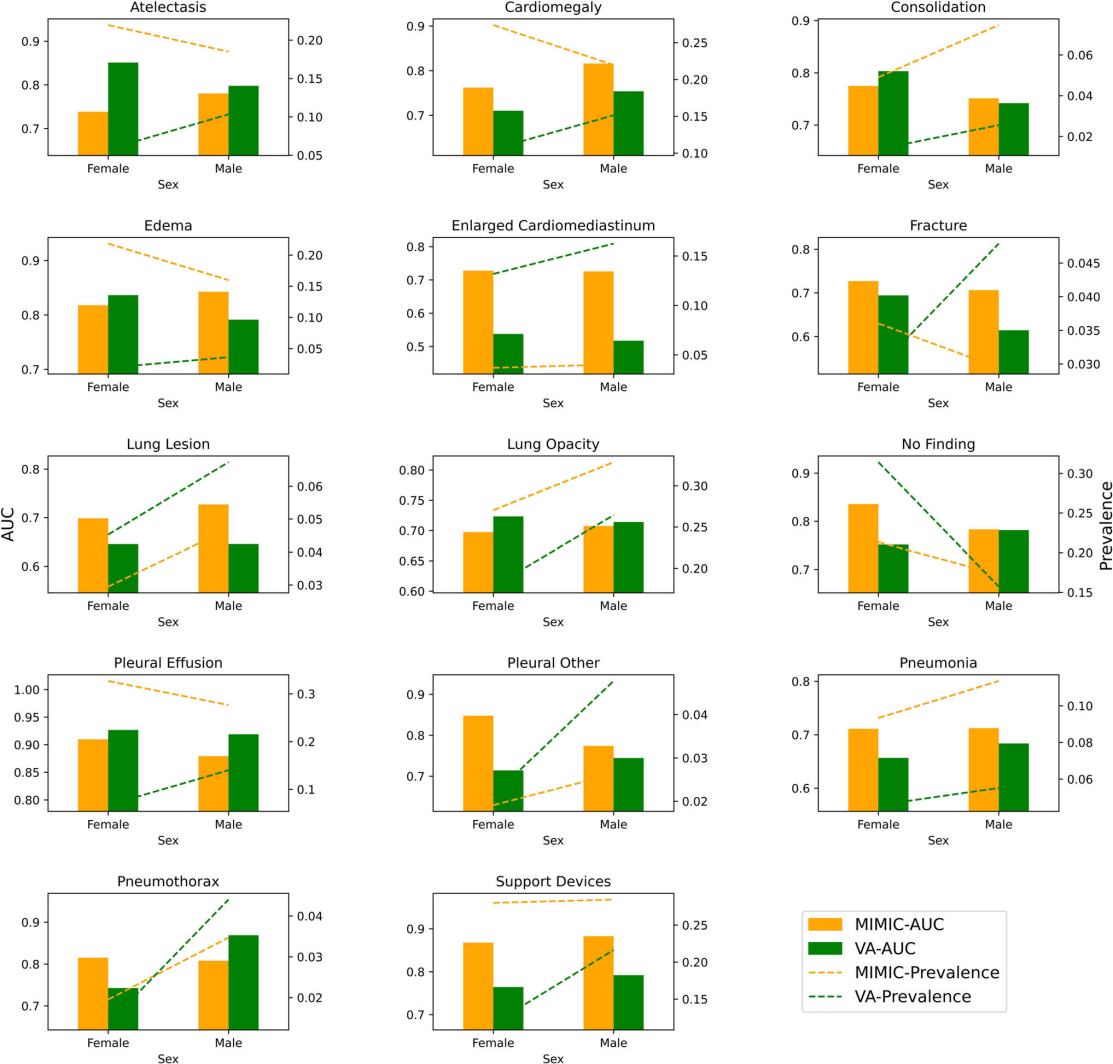
Comparison of label-wise AUC and prevalence across the sexes for MIMIC-CXR and VA-CXR. Orange represents MIMIC-CXR, and green represents VA-CXR

Figure 4 compares the label-wise AUC and prevalence of the two sexes across the Test Split MIMIC-CXR and VA-CXR. This comparison is essential as the female-male patient ratio in VA-CXR is higher than that of MIMIC-CXR; dashed lines in Fig. 4 can observe this. Based on the *p*-value calculated using the AUC two-tailed z-test, it was observed statistically significant difference in performance between MIMIC-CXR and VA-CXR for male population for the labels: enlarged cardiomediastinum (*p*-value: 0.002092) and support devices (*p*-value: 0.003671), consistent with Table 4.

### View Position-Wise Across Datasets

**Fig. 5:**
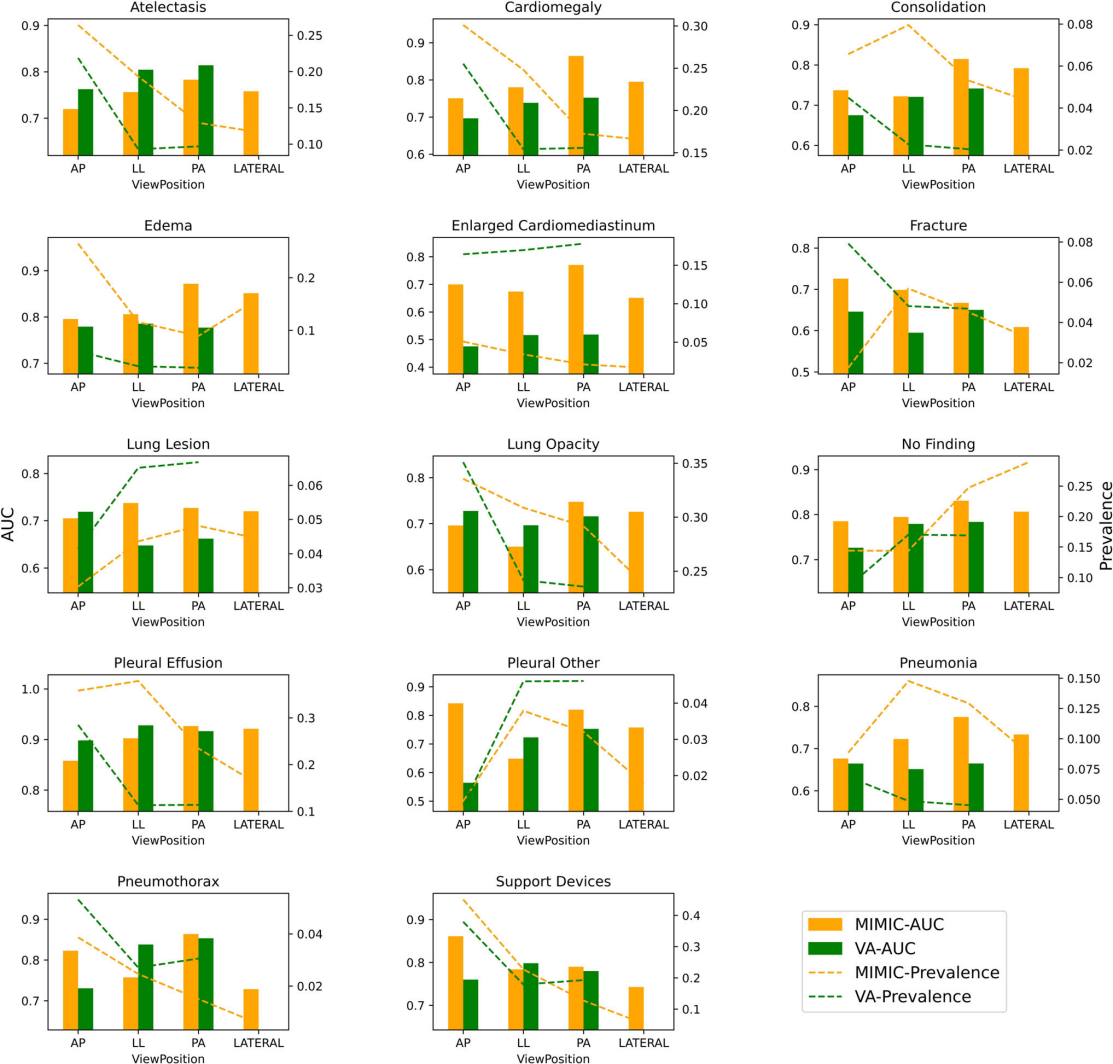
Comparison of AUC and prevalence across view points

Figure 5 compares labels across Test Split MIMIC-CXR and VA-CXR. The VA-CXR does not contain any *lateral* images; the figure shows only MIMIC-CXR performance on the *lateral*. The prevalence and AUC of viewpoints vary based on the label, with *ECM* having the most difference between the datasets. *Pleural other* has a drop in performance in VA-CXR in *AP* view position, potentially due to low prevalence in both datasets. Based on the *p*-value calculated using the AUC two-tailed z-test, it was observed that there were no significant statistical differences in performance between MIMIC-CXR and VA-CXR.

### Age Group-Wise Comparison Across Datasets

**Fig. 6:**
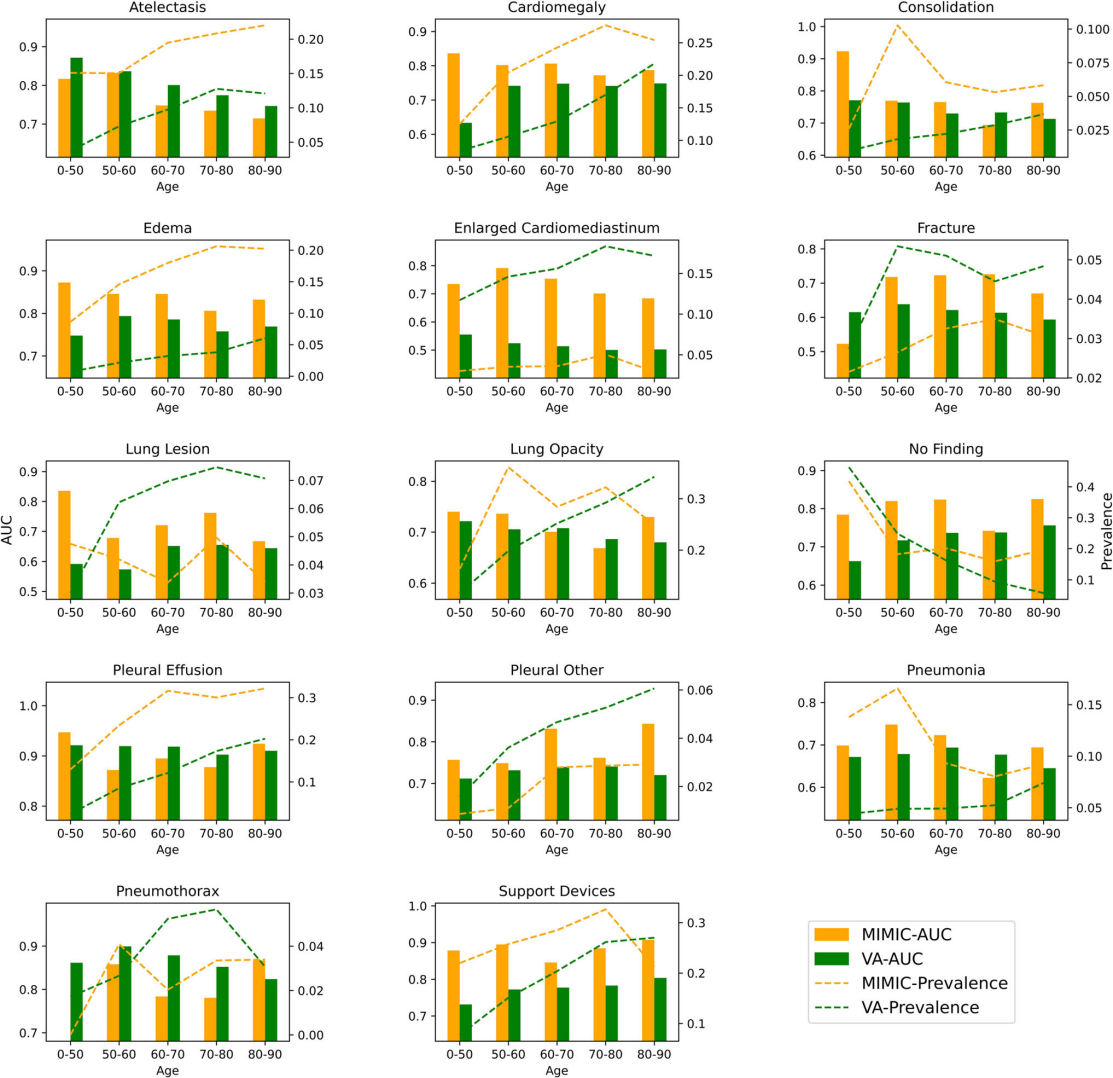
Age group label-wise distribution

Figure 6 shows the VA-CXR prevalence increase as the age increases across all labels except *no finding*, *lung lesion*, and *pneumothorax*. The highest performance drops of 0.15 to 0.2 AUC in VA-CXR compared to the Test Split of MIMIC-CXR can be observed in *enlarged cardiomediastinum* and *support devices*. In VA-CXR, it is interesting that the AUC performance across the age groups is more stable, i.e., there is not much change in AUC, with the exception of *atelectasis*.

### Summary

As seen from the results, the ground truth validation and multi-label classification performance across the NLP extraction tools showed that though the VA-CXR dataset has lesser disagreement rates than the MIMIC-CXR datasets, there were AUC differences between models when using CheXpert and CheXbert (potentially propagated by distribution differences). When comparing the multi-label classification performance on different datasets, the unseen datasets did not show domain shift other than a few labels such as *enlarged cardiomediastinum*. Among the different subgroup analyses, the study year had the most drastic differences in the performance of the multi-label classification model. These differences indicate that the domain shift is definitely of concern in study years.

## Discussion

Our study quantified the domain shift between a open critical care dataset (MIMIC-CXR) and an outpatient, private dataset (VA-CXR), assessing the efficacy of transfer learning for automated chest X-ray classification. Because domain shift encompasses multifaceted factors, our comprehensive study was structured into three interrelated parts: the quality of ground truth, performance comparison, and subgroup analysis.

### Quality of Ground Truth

Supervised learning models rely heavily on the quality of labels available for training. Therefore, we quantified the ground truth quality extracted from radiology reports in both datasets. Despite being a source dataset, MIMIC-CXR exhibited significantly higher disagreement rates between the two ground truth information extraction NLP algorithms than the target, VA-CXR dataset. The potential performance drift of the target can be attributed to the mismatch of ground truth extraction methods. Our analysis underscored the importance of high-quality annotations in mitigating the effects of domain shift [16, 17] and improving model performance.

### Performance Comparison

We compared the difference in classification performance between MIMIC-CXR and VA-CXR to understand the extent of domain shift and its implications for model generalization. Our findings revealed notable variability in classification accuracy, highlighting the challenges posed by domain shift. We observe that the prevalence and performance were directly associated; for example, MIMIC-CXR’s *enlarged cardiomediastinum* low prevalence may be the reason for its low performance.

### Subgroup Analysis

Subgroup analysis using demographic factors is crucial as we expect our populations to have different demographic distributions [18]. We observed a decline in the performance of the VA-CXR dataset over time, particularly in the years following 2020. This drop may be due to differences in the study years between the source dataset (MIMIC-CXR) and the VA-CXR dataset, as well as potential impacts from the pandemic years (note: we did not evaluate additional labels related to the coronavirus pandemic). We observed that both groups, though with different distributions, have aging populations across conditions of interest. Still, the prevalence of most conditions with age increases in the VA-CXR dataset, but it is highly variable in MIMIC-CXR. For sex subgroup analysis, the performance was similar across the female and male populations in the datasets. Though the VA-CXR population is skewed towards the male population, the model performed well on the female population. This can be attributed to the source domain (MIMIC-CXR) having a balanced male-to-female ratio. For the view-based subgroup, we observed high variability between the views. This behavior can be attributed to the necessity of specific views for accurately diagnosing certain diseases. For instance, conditions such as pneumonia and ECM primarily rely on PA (posteroanterior) or AP (anteroposterior) views for diagnosis.

### Clinical Relevance

Despite the surge in artificial intelligence and machine learning applications in clinical settings, their adoption has been uneven, primarily due to disparities in funding, expertise, and availability of computing resources [19]. While large academic medical centers have been at the forefront of AI adoption, the accessibility of such resources remains a challenge for smaller research institutions. Introducing transfer learning has alleviated some of these concerns by enabling researchers to leverage pre-existing models trained on open datasets for their specific applications. But transfer learning approaches need to be evaluated for domain shift that poses a significant obstacle to achieving robust and generalizable models.

For example, pre-trained deep learning models that are generalizable can be used/adapted for automated medical imaging analysis in low-resource settings. These models will help address the global shortage of radiologists by enhancing workflow efficiency and reducing diagnostic delays, particularly in regions where access to expert interpretation is limited. However, to ensure real-world applicability, transfer learning approaches will need to overcome domain shifts caused by variations in patient demographics, imaging equipment, and hospital protocols, which can significantly impact model performance. Addressing these challenges through domain adaptation techniques, federated learning, and continuous model monitoring will be critical to make AI-driven diagnostics more reliable and equitable in diverse clinical environments. In addition, integrating explainability features into AI models will be essential to gain clinician trust and ensure regulatory compliance, further facilitating their adoption in medical practice.

### Potential Workflows

While we primarily focused on matching image classification to known findings typical of chest X-rays and extracted those from radiology reports using NLP, this may not be the best workflow with which image classification can be incorporated into a medical outcomes workflow. Tiu et al. have noted the difficulties and inaccuracies associated with training chest X-ray classifiers against such labels and demonstrated improved accuracy and efficiencies with a self-supervised workflow to train their image classification model [20]. Even more ambitiously, they argue in Reference [21] that all modalities of data should be co-optimized in a much broader framework, known as a foundation model. Our observations in Fig. 2 show that most of the X-ray findings do not map directly onto diagnoses support the idea that more flexible workflows may be required to optimally incorporate AI-based image classification into medical outcome prediction.

By systematically addressing the challenges of domain shift and demographic factors, our study contributes valuable insights to developing more accurate and robust chest X-ray classification models. Furthermore, our findings have broader implications for healthcare, emphasizing the importance of understanding and mitigating biases in machine learning applications to ensure equitable healthcare delivery. Our research serves as a foundation for future studies aimed at refining transfer learning techniques and enhancing the generalizability of machine learning models in clinical practice.

### Limitations

#### Datasets

The images from two datasets were collected in significantly different clinical settings, likely influencing the outcomes. The MIMIC database primarily includes critical care inpatient data, characterized by a prevalence of portable anteroposterior (AP) images that tend to be less precise. In contrast, the VA outpatient population consists of individuals who are generally less acutely ill, with imaging conducted in a controlled outpatient setting, potentially resulting in higher-quality images. Although we did not conduct a quantitative assessment of these differences, we hypothesize that variations in clinical settings, disease severity, and imaging protocols could substantially impact model performance.

#### Classification Models

Our decision to primarily use DenseNet121 was guided by its strong performance in prior literature [12, 22] and its computational efficiency, which is particularly relevant in resource-constrained hospital environments. While we also evaluated a category-wise fine-tuning (CFT) ensemble method, future work should explore additional architectures, such as transformer-based models or hybrid ensemble approaches, to improve robustness against domain shifts.

#### Imaging Protocols, Preprocessing, and Label Noise

Variations in imaging protocols, equipment, and radiologist expertise contribute to domain shift, impacting model performance. While we attempted to mitigate some of these effects through image resizing during preprocessing, differences in acquisition parameters and manufacturer-specific imaging characteristics may still introduce inconsistencies. This challenge is particularly pronounced in modalities like MRI, where hardware-specific variations necessitate extensive preprocessing adjustments.

Additionally, ground truth label quality is affected by inconsistencies in radiology reporting styles across institutions, clinics, and geographic regions. Since our study relies on NLP-extracted labels from radiology reports, variations in terminology and reporting standards may introduce label noise. Our analysis revealed notable differences between CheXpert and CheXbert labelers, with disagreement rates varying across labels. For example, cardiomegaly in MIMIC-CXR had a 39.1% disagreement rate, and in VA-CXR, it was at 13.7%. These levels of disagreement between NLP labelers highlight the inherent limitations of automated labeling methods.

Furthermore, the role of preprocessing techniques—such as image normalization, resolution adjustments, and harmonization strategies—was not explicitly quantified in this study. Future work should include ablation studies to evaluate their impact on mitigating domain shifts. Additionally, integrating structured reporting guidelines and harmonization techniques may improve cross-domain generalizability and label consistency.

#### Manual Annotation and Expert Review

The absence of manual annotations as a gold standard remains a limitation. While NLP-based label extraction enables scalability, it is susceptible to errors arising from ambiguous phrasing or missing information in radiology reports as discussed earlier. Future work should incorporate expert-reviewed subsets to strengthen validation and improve the reliability of ground truth labels.

One promising approach is the integration of a human-in-the-loop framework, where radiologists provide active feedback on model predictions to refine label accuracy over time. Additionally, retrieval-augmented generation (RAG) techniques could be explored to enhance label extraction by combining NLP-generated insights with expert validation. By leveraging these strategies, future studies could develop more robust models that better account for label noise and improve generalizability.

## Conclusion

This work provides valuable insights into the interplay between domain shift, demographic factors, and the quality of ground truth annotations in automated image classification tasks. By demonstrating the limitations of current transfer learning approaches, we emphasize the need for more equitable and robust machine learning models in clinical practice. The broader impact of this research lies in its potential to guide the development of AI systems that can generalize effectively across diverse clinical settings, thereby reducing biases and improving healthcare outcomes.

### Future Directions

Building on this foundation, future research should focus on integrating manual annotations to establish a reliable gold standard for ground truth labels, enhancing the robustness of transfer learning techniques through advanced methods like domain adaptation and self-supervised learning and exploring a wider range of deep learning models and ensemble strategies. Additionally, investigating the impact of imaging protocols, radiologist expertise, and equipment variability with quantitative metrics will provide a more comprehensive understanding of domain shift. Finally, efforts to develop datasets that reflect a wide range of patient demographics to ensure fair healthcare outcomes and reduce disparities in AI-driven diagnostics.

## Figures and Tables

**Fig. 7:**
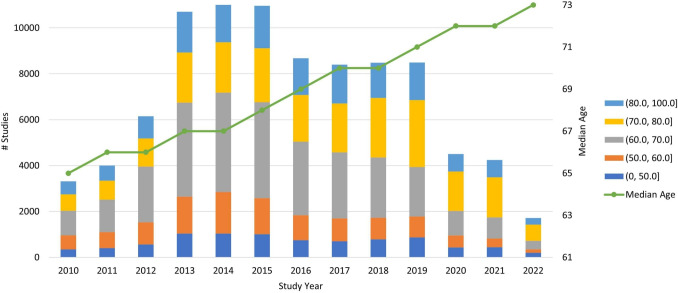
Study year vs age when image was taken

## Appendix

### Age When Image Was Taken Versus Study Year

**Table 5:** View position distribution between datasets

	Source dataset		Target dataset	
Name	MIMIC-CXR		VA-CXR	
View position	# Images	%	# Images	%
Anterior/posterior (AP)	147,169	39.02	879	0.34
Posterior/anterior (PA)	96,155	25.5	52,749	20.33
Lateral	82,852	21.97	11	–
Left lateral (LL)	35,129	9.31	50,267	19.38
Others	23	–	6	–
Unknown	15,769	4.18	159,482	61.49

**Table 6:** Groundtruth validation using ICD codes

Finding	ICD-9 codes	ICD-10 codes
Pneumonia	480 to 486, 487.0	J10.0, J11.0, J2 to J8
Atelectasis	518.0, 770.4	J98.11
Cardiomegaly	429.3	I51.7
Edema	518.4, 514, 428	J81, I50
Enlarged cardiomediastinum	519.2	J98.5
Fracture	807.[0to4], 806.[2to3]	S22
Lung lesion	793.11	R91
Lung opacity	516	J84
Pleural effusion	511.[1,8,9]	J90, J91
Pleural other	511.0, 515	J92, J94, J84.[0,1]
Pneumothorax	512.[0,1,8], 512.8[1,2,3,9]	J93

**Table 7:** Groundtruth validation using ICD codes count

Findings	ICD Dx w.r.t Img	Uncertain		Negative		Positive		Null	
		Bert	Pert	Bert	Pert	Bert	Pert	Bert	Pert
Pneumonia	ICD Dx <1 wk	841	762	248	91	1336	1590	2808	2790
	ICD Dx > 1 wk	1895	1546	1366	675	1715	2821	29,002	28,936
	No Dx	1261	1018	1657	965	564	1571	51,964	51,892
Atelectasis	ICD Dx < 1wk	146	138	3	2	415	424	363	363
	ICD Dx > 1 wk	692	680	9	15	1366	1375	5068	5065
	No Dx	3983	3879	45	83	7573	7626	71,794	71,807
Cardiomegaly	ICD Dx < 1wk	59	51	82	54	469	477	187	215
	ICD Dx > 1 wk	887	821	2098	1499	1889	2220	3651	3985
	No Dx	6121	5745	31,441	22,643	6925	10,967	37,627	42,759
Edema	ICD Dx 90 days	2054	2154	6859	6247	1677	2057	8271	8403
	ICD Dx > 90 days	2456	2629	13,590	12,653	1806	2312	16,886	17,144
	No Dx	833	973	20,304	19,353	403	702	32,598	33,110
ECM	ICD Dx < 1wk	32	23	5	3	7	17	54	55
	ICD Dx > 1 wk	136	78	48	44	29	96	266	261
	No Dx	23,755	14,535	15,744	3992	3091	14,576	47,924	47,411
Fracture	ICD Dx < 1wk	0	1	2	0.5	18	18	297	298
	ICD Dx>1 wk	1	1	38	24	238	244	4278	4286
	No Dx	96	152	962	652	3913	4029	81,262	81,401
Lung lesion	ICD Dx < 1wk	5	5	29	14	162	168	4251	4260
	ICD Dx > 1 wk	37	52	308	185	1502	1546	30,315	30,379
	No Dx	60	101	692	490	2632	2708	54,768	54,853
Lung opacity	ICD Dx < 1wk	0	31	60	46	301	424	342	202
	ICD Dx > 1 wk	10	246	606	761	1852	2444	3131	2148
	No Dx	178	2748	11,179	18,518	16,136	21,049	57,794	42,972
Pleural effusion	ICD Dx < 1wk	201	277	326	276	3127	2970	619	750
	ICD Dx > 1 wk	518	1098	5580	5080	5343	5060	2971	3174
	No Dx	1238	4015	55,570	52,665	6719	6665	12,141	12,323
Pleural other	ICD Dx < 1wk	83	71	0.5	2	352	307	3838	3893
	ICD Dx > 1 wk	212	216	1	9	1270	1089	12,929	13,098
	No Dx	422	462	11	31	3710	3109	71,525	72,066
Pneumothorax	ICD Dx < 1wk	57	42	303	265	782	834	139	140
	ICD Dx > 1 wk	59	114	1168	1037	775	836	1556	1571
	No Dx	250	1871	35,272	32,893	2317	2739	49,095	49,431

*ECM*, enlarged cardiomediastinum; *Dx*, diagnosis; *ICD Dx w.r.t Img*, ICD diagnose code with respect to the date of imaging study
